# Supplementary material for: Cancer immune therapy with PD-1-dependent CD137 co-stimulation provides localized tumour killing without systemic toxicity
Source: Nat Commun. 2021 Nov 4;12:6360. doi: 10.1038/s41467-021-26645-6 (PMC8569200; doi:10.1038/s41467-021-26645-6)
Supplement: Supplementary file 1 — Supplementary Information [file 41467_2021_26645_MOESM1_ESM.pdf]

Supplementary Figures and tables for:

Cancer immune therapy with PD-1-dependent CD137 co-stimulation provides localized tumour killing without systemic toxicity

Yunqian Qiao[1#], Yangmin Qiu[1#], Jie Ding[1], Nana Luo[1], Hao Wang[1], Xiaomin Ling[1], Jiya Sun[1], Zhihai Wu[1], Yisen Wang[1], Yanpeng Liu[1], Feifei Guo[1], Ta Sun[1], Wanwan Shen[1], Min Zhang[1], Dongdong Wu[1], Bingliang Chen[1], Wei Xu[1*], Xuan Wang[1*]

[1]Innovent Biologics (Suzhou) Co. Ltd, Suzhou, China
[#]Equally contributing authors
*Corresponding authors: Wei Xu: xu.wei@innoventbio.com, Xuan Wang: xuan.wang@innoventbio.com

a

Name	Fc	K_D (nM, BLI)	
		hPD-1	hCD137
IBI319	silenced	0.632±0.059	719±214
αPD-1		1.14±0.090	n.a.
αCD137		n.a.	462±130

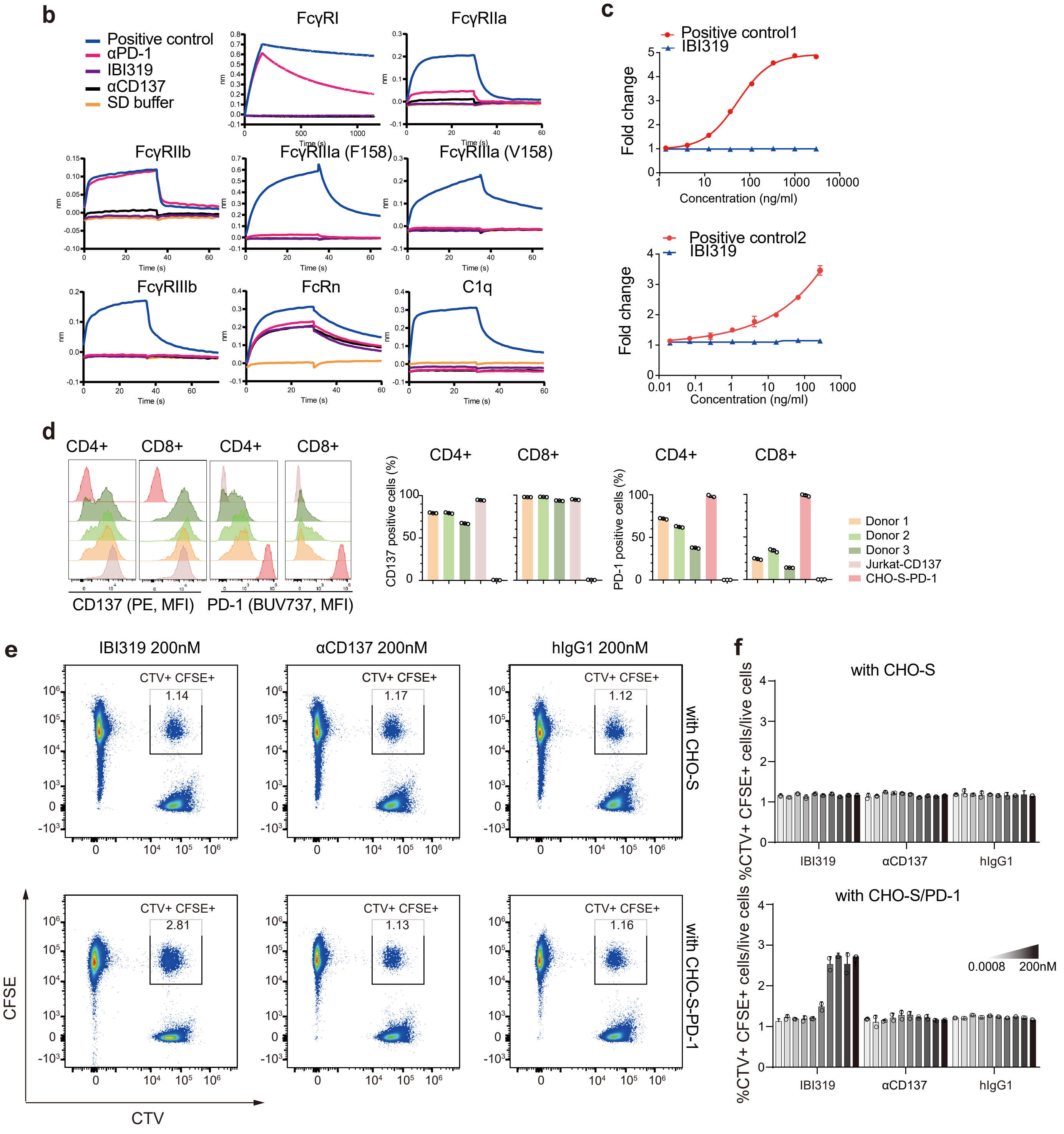

Supplementary Fig. 1

a The dissociation constants (KDs) of IBI319, αPD-1, and αCD137 for human PD-1 and human CD137, respectively, determined via BLI (mean SD; n.a. not analysed). (**b**, **c**) Fc receptor binding affinity and effector function of IBI319. **b** Association and dissociation of IBI319, αPD-1 and αCD137 with FcγRs, FcRn and C1q measured by ForteBio-based SPR (one representative experiment from 2 biological replicates). **c** ADCC activity of IBI319 mediated by the anti-PD-1 arm (top) or anti-CD137 arm (bottom) tested in a luciferase-based ADCC functional assay (mean and SD of 2 technical replicates of one representative experiment from 2 biological replicates). An IgG1 antibody served as a positive control in the two assays. Error bar: SD. **d** PD-1 and CD137 expression on CD4+ and CD8+ T cells from 3 healthy donors and comparison with the Jurkat-CD137 and CHO-S-PD-1 cell lines, evaluated by flow cytometry (FACS). (Left) FACS histogram (right) Quantification of the CD137+ and PD-1+ cell proportions; n = 3 technical replicates, mean and SD. (**e**, **f**) IBI319 can bridge Jurkat-CD137 and CHO-S-PD-1 cells. **e** The percentage of the CTV and CFSE double-positive population in different treatment groups determined via FACS. Jurkat-CD137 cells were characterized as CTV+, and CHO-S-PD-1 or CHO-S cells were characterized as CFSE+. **f** Quantification of the CTV and CFSE double-positive cell percentage in live cells in different treatment groups. n = 2 technical replicates; Mean and SD.

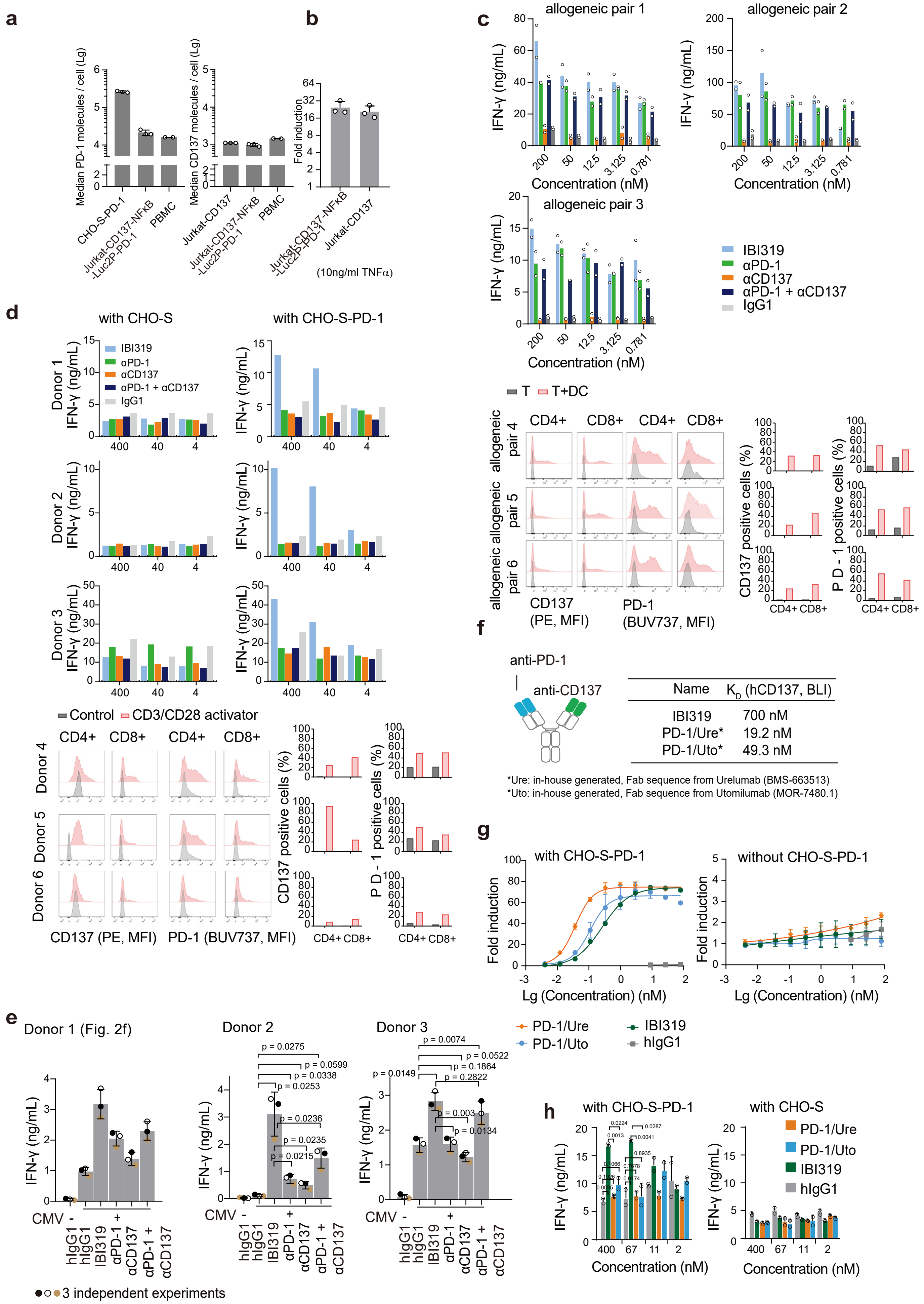

Name	K_D (hCD137, BLI)
IBI319	700 nM
PD-1/Ure*	19.2 nM
PD-1/Uto*	49.3 nM

*Ure: in-house generated, Fab sequence from Urelumab (BMS-663513)
*Uto: in-house generated, Fab sequence from Utomilumab (MOR-7480.1)

Supplementary Fig. 2

a QIFIKIT quantification of cell-surface receptors on cell lines and PBMC-derived CD3+ T cells stimulated by CD3/CD28 activator (strong stimulation: 25 μL/mL) (n = 2 independent experiments for PBMC or n = 3 independent experiments for other cells; Mean and SD). **b** Jurkat-CD137-NFκB-Luc2P-PD-1 and Jurkat-CD137 cells harbour similar NFκB activation potentials after TNFα stimulation (3 independent experiments; mean and SD). **c** IFN-γ production level (top) and PD-1 and CD137 expression analyses (bottom, separate donor pairs) in an MLR assay evaluating 3 allogenic T cell-DC pairs. Antibodies were 4-fold serially diluted from 200 nM (n = 2 technical replicates). **d** IFN-γ production level in a T cell co-stimulation assay evaluating 3 donors (top) and PD-1 and CD137 expression analyses in 3 separate donors after sub-optimal CD3/CD28 activator stimulation (1 μL/mL) (bottom). Antibodies were serially diluted 10-fold from 400 nM in the presence of CHO-S or CHO-S-PD-1 cells. **e** IFN-γ production level in a CMV T cell memory recall assay. PBMCs from 3 donors previously infected with CMV were co-cultured with a CMV peptide pool and antibodies at 50 nM for 4 days. Data show individual values of 3 independent experiments and the mean ± SD). (**f-h**) A comparison of IBI319 and two other CD137/PD-1 bispecific antibodies. The anti-CD137 arm of IBI319 was replaced by in-house-generated monovalent urelumab (Ure) or utomilumab (Uto) to generate PD-1/Ure and PD-1/Uto, respectively. **f** Cartoon of the bispecific antibodies (left) and the binding affinities of IBI319, PD-1/Ure and PD-1/Uto for CD137 determined using BLI (right). **g** CD137 agonist activities of different antibodies presented as NFκB-mediated luciferase activity. Jurkat-CD137-NFκB-Luc (Jurkat-CD137) cells were co-cultured with or without 2 times the number of CHO-S-PD-1 cells. Data show the mean ± SD of two technical replicates of one representative experiment from 3 independent experiments. **h** IFN-γ production level in a T cell co-stimulation assay. Primary T cells were treated with 1 μL/mL CD3/CD28 activator, and antibodies were serially diluted 6-fold from 400 nM in the presence of CHO-S or CHO-S-PD-1 cells. The bar plot shows the mean and SD of results from 2 donors. Error bar: SD. Statistics were performed in the indicated groups: two-sided, paired *t*-test (**e**) and two-sided, unpaired *t*-test (**h**); *p* values are indicated in the graphs.

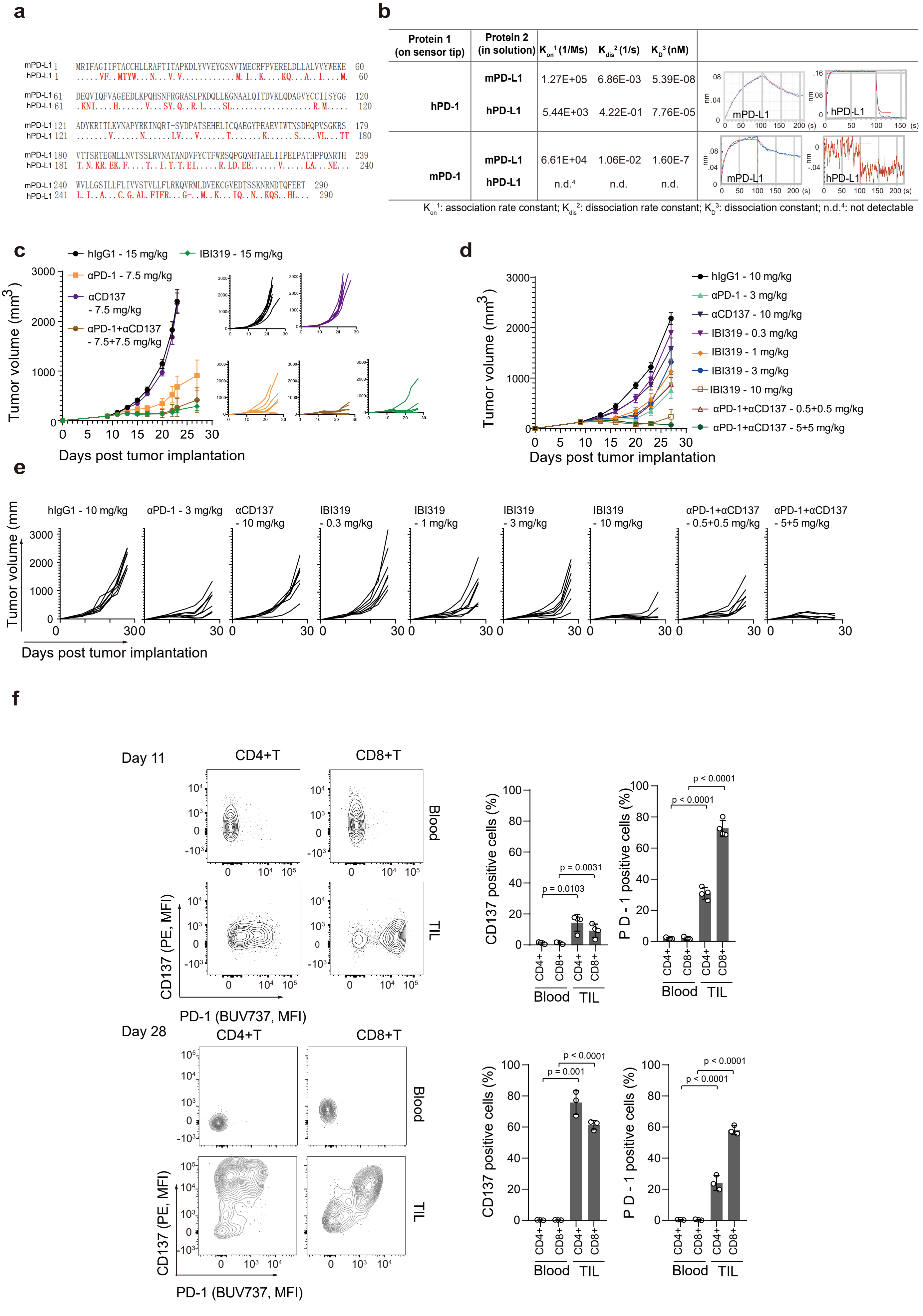

(to be continued on the next page)

g

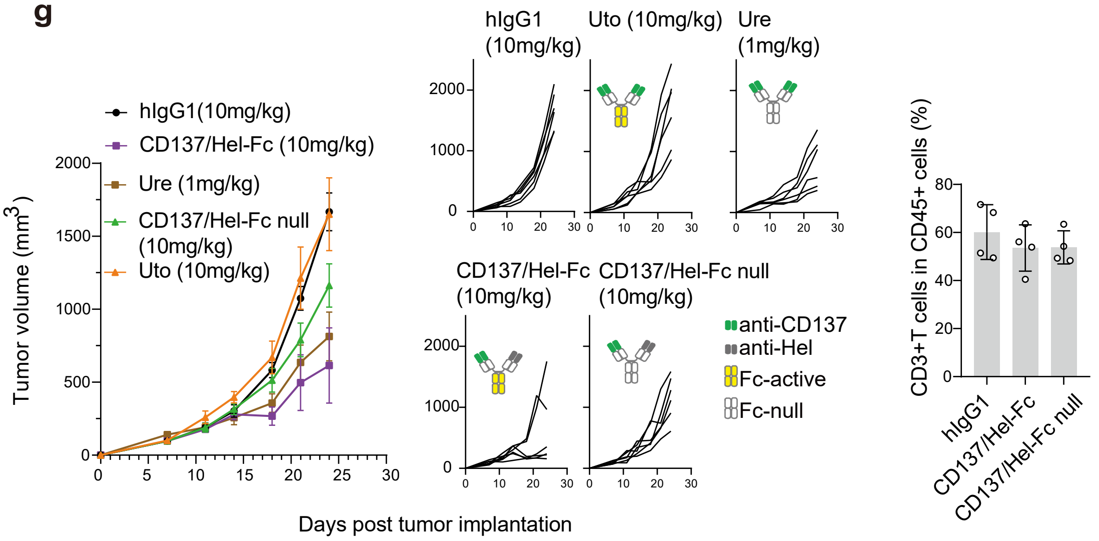
hIgG1(10mg/kg)
CD137/Hel-Fc (10mg/kg)
Ure (1mg/kg)
CD137/Hel-Fc null (10mg/kg)
Uto (10mg/kg)
Tumor volume (mm^3)
Days post tumor implantation
hIgG1 (10mg/kg)
Uto (10mg/kg)
Ure (1mg/kg)
CD137/Hel-Fc (10mg/kg)
CD137/Hel-Fc null (10mg/kg)
anti-CD137
anti-Hel
Fc-active
Fc-null
CD3+T cells in CD45+ cells (%)
hIgG1
CD137/Hel-Fc
CD137/Hel-Fc null

h

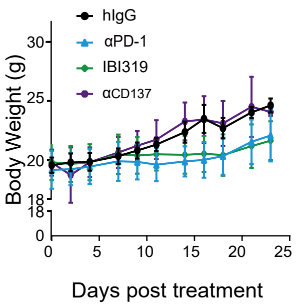
hIgG
αPD-1
IBI319
αCD137
Body Weight (g)
Days post treatment

i

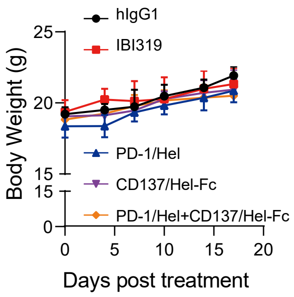
hIgG1
IBI319
PD-1/Hel
CD137/Hel-Fc
PD-1/Hel+CD137/Hel-Fc
Body Weight (g)
Days post treatment

j

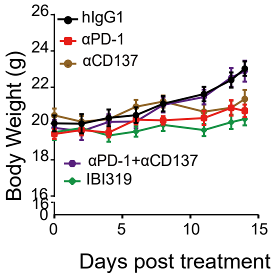
hIgG1
αPD-1
αCD137
αPD-1+αCD137
IBI319
Body Weight (g)
Days post treatment

k

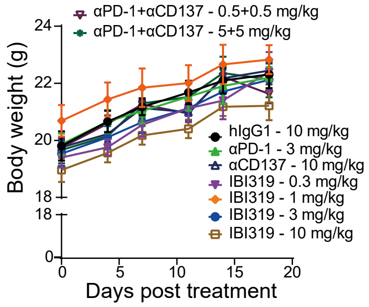
αPD-1+αCD137 - 0.5+0.5 mg/kg
αPD-1+αCD137 - 5+5 mg/kg
hIgG1 - 10 mg/kg
αPD-1 - 3 mg/kg
αCD137 - 10 mg/kg
IBI319 - 0.3 mg/kg
IBI319 - 1 mg/kg
IBI319 - 3 mg/kg
IBI319 - 10 mg/kg
Body weight (g)
Days post treatment

l

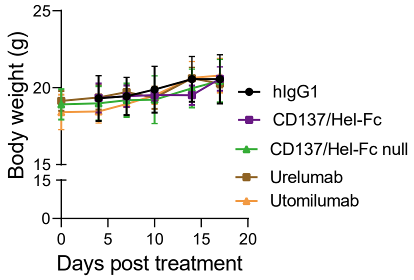
hIgG1
CD137/Hel-Fc
CD137/Hel-Fc null
Urelumab
Utomilumab
Body weight (g)
Days post treatment

Supplementary Fig. 3

a Alignment analysis of murine PD-L1 (mPD-L1; *Mus musculus* strain C57BL/6J, GenBank: GQ904196) and human PD-L1 (hPD-L1; *Homo sapiens*, GenBank: BC069381.1). **b** Binding affinities of hPD-1 and mPD-1 for hPD-L1 and mPD-L1, respectively, measured by BLI. **c** (Left) Tumour volumes of CT26-hPD-L1 tumours in BALB/c-hPD-1/hPD-L1/hCD137 mice treated with the indicated antibodies on days 9, 16, and 23 post tumour implantation (n = 6 per group, error bar: SEM). The mice in the hIgG1 and αCD137 groups were euthanized on day 25 post tumour implantation due to ethical issues. (Right) Spaghetti plots show individual tumour volumes in each group. **d** Tumour volumes of MC38 tumours in C57BL/6-hPD-1/hCD137 mice treated with the indicated antibodies (n = 7 per group, completed groups of the experiment shown in Fig. 3b, error bar: SEM). **e** Individual tumour growth spaghetti plots for the data in **d**. **f** PD-1 and CD137 expression on CD4+ and CD8+ T cells from the blood and TIL samples on day 11 and day 28 post tumour implantation of the hIgG1 control group in Fig. 3b, tested by FACS. Left: Representative FACS plot; right: quantification of CD137+ and PD-1+ cells in CD4+ and CD8+ T cells (n = 4 mice per group for day 11 samples, n = 3 mice per group for day 28 samples; Mean and SD, Statistics were performed in the indicated groups: two-sided, unpaired *t*-test; *p* values are indicated in the graphs.). **g** Tumour volumes of MC38 tumours in C57BL/6-hPD-1/hCD137 mice treated with hIgG1 or the indicated CD137-specific antibodies (1 mg/kg for Ure and 10 mg/kg for all other groups) on days 7, 14 and 21 (n = 6 per group) (left) and individual tumour growth spaghetti plots (middle). Analysis of CD3+T cells in blood by FACS (right) (n = 4 mice per group; mean and SD). (**h-l**) The body weights of mice in the experiments shown in Fig. 3a and e and Supplementary Fig. 3c, d, and g, respectively (**h** n = 8 per group, mean and SD; **i** n = 6 per group for the IgG1 and αPD-1/Hel + αCD137/Hel-Fc groups; n = 5 per group for all other groups, mean and SD; **g** n = 6 per group, mean and SD; **k** n = 7 per group, mean and SD; **l** n = 6 per group, mean and SD).

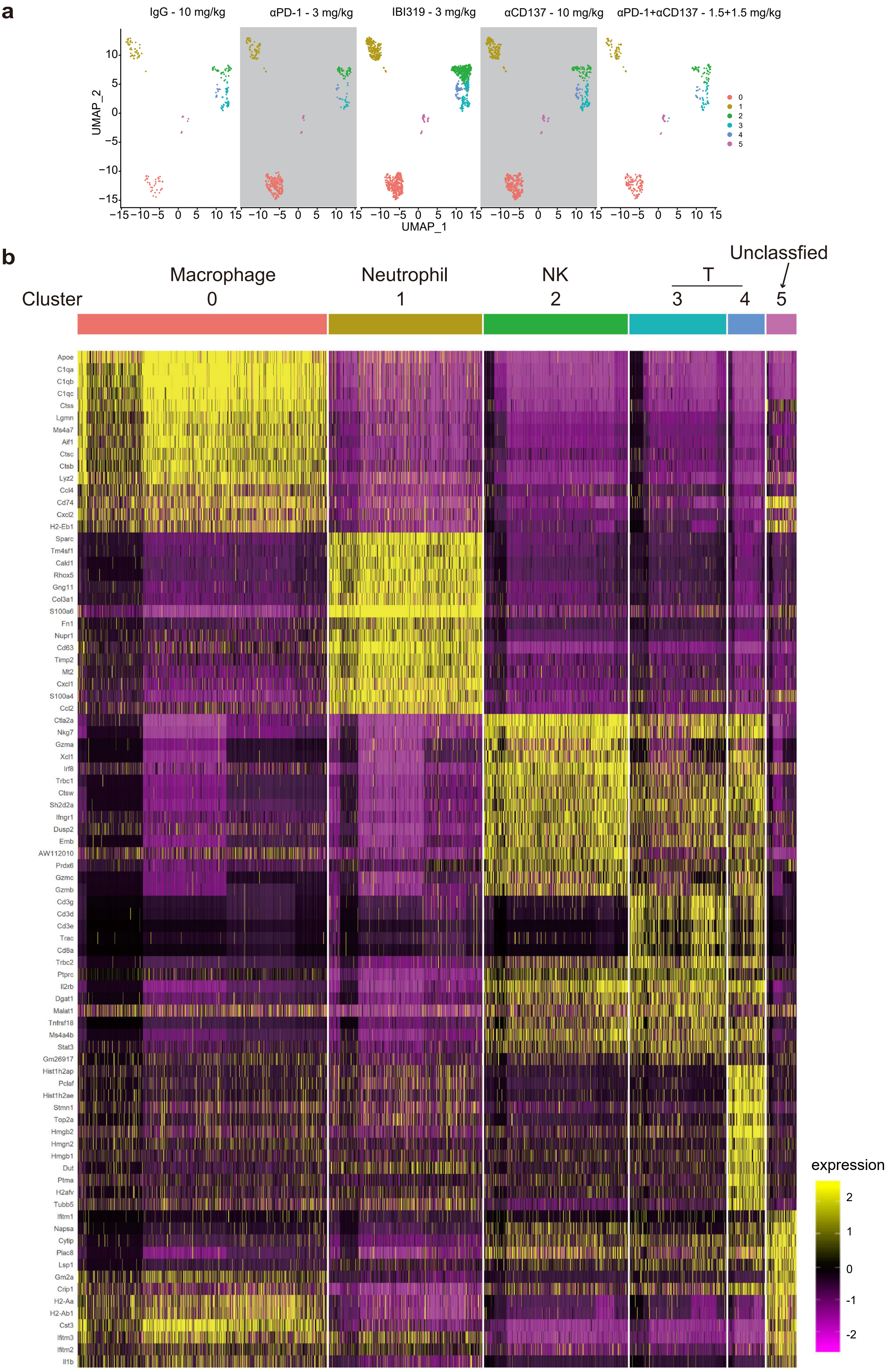
a
IgG - 10 mg/kg
αPD-1 - 3 mg/kg
IBI319 - 3 mg/kg
αCD137 - 10 mg/kg
αPD-1+αCD137 - 1.5+1.5 mg/kg
UMAP_2
UMAP_1
0
1
2
3
4
5
b
Cluster
Macrophage
0
Neutrophil
1
NK
2
T
3
4
Unclassfied
5
Apoe
C1qa
C1qb
C1qc
Ctss
Lgmn
Ms4a7
Aif1
Ctsc
Ctsb
Lyz2
Ccl4
Cd74
Cxcl2
H2-Eb1
Sparc
Tm4sf1
Cald1
Rhox5
Gng11
Col3a1
S100a6
Fn1
Nupr1
Cd63
Timp2
Mt2
Cxcl1
S100a4
Ccl2
Ctla2a
Nkg7
Gzma
Xcl1
Irf8
Trbc1
Ctsw
Sh2d2a
Ifngr1
Dusp2
Emb
AW112010
Prdx6
Gzmc
Gzmb
Cd3g
Cd3d
Cd3e
Trac
Cd8a
Trbc2
Ptprc
Il2rb
Dgat1
Malat1
Tnfrsf18
Ms4a4b
Stat3
Gm26917
Hist1h2ap
Pclaf
Hist1h2ae
Stmn1
Top2a
Hmgb2
Hmgn2
Hmgb1
Dut
Ptma
H2afv
Tubb5
Ifitm1
Napsa
Cytip
Plac8
Lsp1
Gm2a
Crip1
H2-Aa
H2-Ab1
Cst3
Ifitm3
Ifitm2
Il1b
expression
2
1
0
-1
-2

Supplementary Fig. 4

a UMAP plots of single CD45+ tumour-infiltrating immune cells derived from 5 groups (hIgG, αPD-1, IBI319, αCD137, and αPD-1+αCD137). Each cluster is represented by a specific colour and number. **b** The expression of signature genes in each cell cluster.

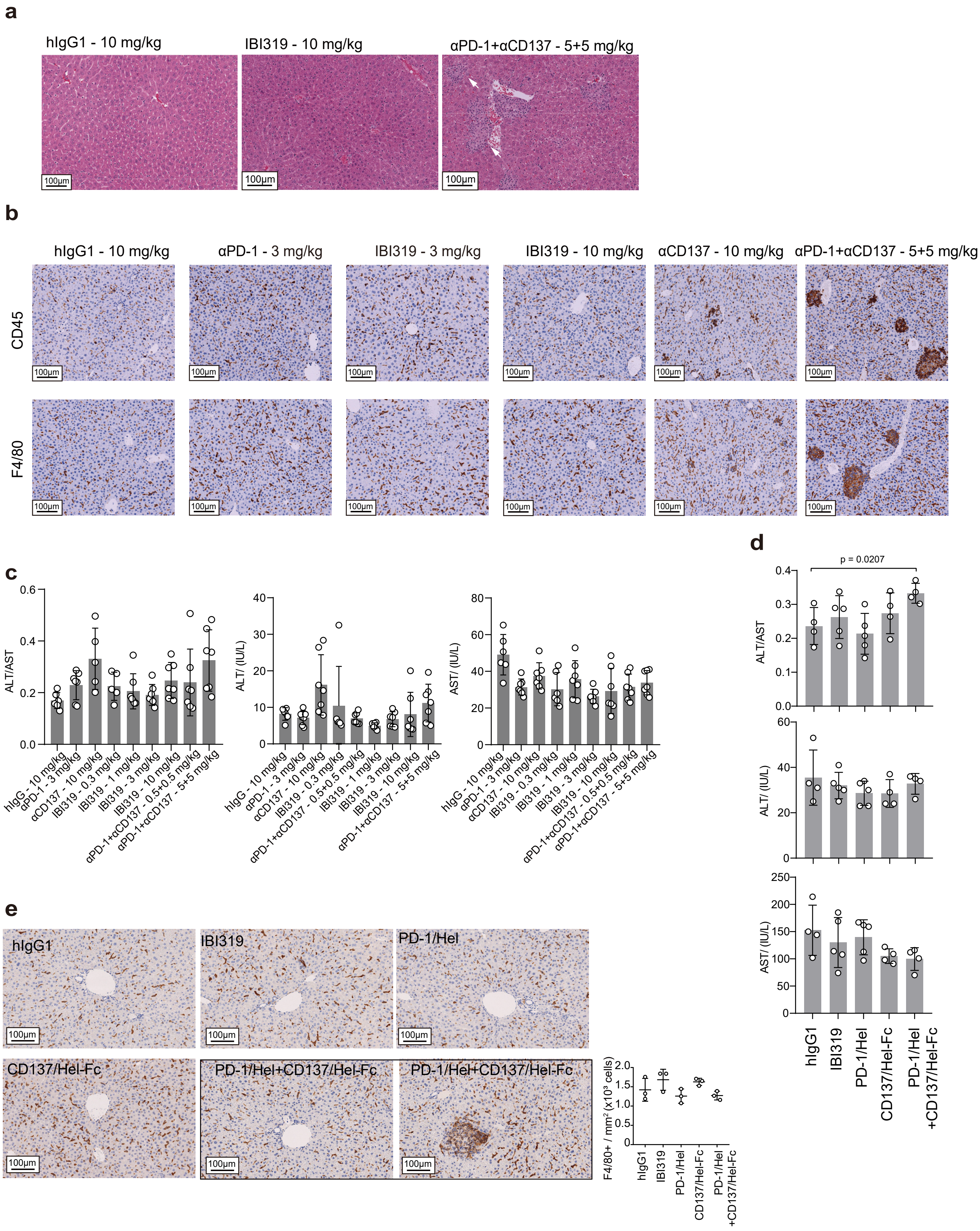

a
hIgG1 - 10 mg/kg
IBI319 - 10 mg/kg
αPD-1+αCD137 - 5+5 mg/kg
100μm
b
hIgG1 - 10 mg/kg
αPD-1 - 3 mg/kg
IBI319 - 3 mg/kg
IBI319 - 10 mg/kg
αCD137 - 10 mg/kg
αPD-1+αCD137 - 5+5 mg/kg
CD45
F4/80
c
ALT/AST
ALT/ (IU/L)
AST/ (IU/L)
hIgG - 10 mg/kg
αPD-1 - 3 mg/kg
αCD137 - 10 mg/kg
IBI319 - 0.3 mg/kg
IBI319 - 1 mg/kg
IBI319 - 3 mg/kg
IBI319 - 10 mg/kg
αPD-1+αCD137 - 0.5+0.5 mg/kg
αPD-1+αCD137 - 5+5 mg/kg
d
p = 0.0207
ALT/AST
ALT/ (IU/L)
AST/ (IU/L)
hIgG1
IBI319
PD-1/Hel
CD137/Hel-Fc
PD-1/Hel +CD137/Hel-Fc
e
hIgG1
IBI319
PD-1/Hel
CD137/Hel-Fc
PD-1/Hel+CD137/Hel-Fc
PD-1/Hel+CD137/Hel-Fc
F4/80+ / mm² (x10³ cells)
hIgG1
IBI319
PD-1/Hel
CD137/Hel-Fc
PD-1/Hel +CD137/Hel-Fc

Supplementary Fig. 5

a Representative H&E staining in the indicated groups. **b** Representative immunohistochemical (IHC) staining for CD45 and F4/80 in all groups of Fig. 3e. **a** and **b** n = 3 mice per group from one experiment were analyzed and the entire slide section of each mouse was scanned with microscope. The image of each group shows a representative area of the entire scan**. c** C57BL/6-hPD-1/hCD137 mouse blood ALT/AST ratios in experiment in Supplementary Fig. 3D (n = 6 per group for hIgG -10 mg/kg and IBI319 - 0.3 mg/kg groups, n = 7 per group for all other groups, mean and SD). **d** C57BL/6-hPD-1/hCD137 mouse blood ALT/AST ratios in experiment in Fig. 3e (n = 5 per group for IBI319 and PD-1/Hel groups, n = 4 for all other groups, mean and SD) (Statistics were performed in the indicated groups: two-sided, unpaired *t*-test; *p* value is indicated in the graphs. **e** Representative IHC staining and quantification for F4/80 in all groups of Fig. 3f (n = 3 per group, mean and SD).

a

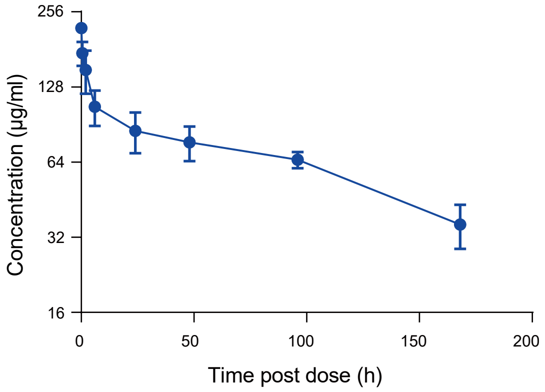

PK parameter	Mean (n=3)
$t_{1/2}$ (h)	111.92
$AUC_{0\text{-}t}$ (h·µg/mL)	11587.55
$AUC_{0\text{-}\infty}$ (h·µg/mL)	17395.38
V_{ss} (mL/kg)	88.94
CL (mL/h/kg)	0.57
$MRT_{0\text{-}t}$ (h)	67.12
C_{max} (µg/mL)	220.15
T_{max} (h)	0.08

b

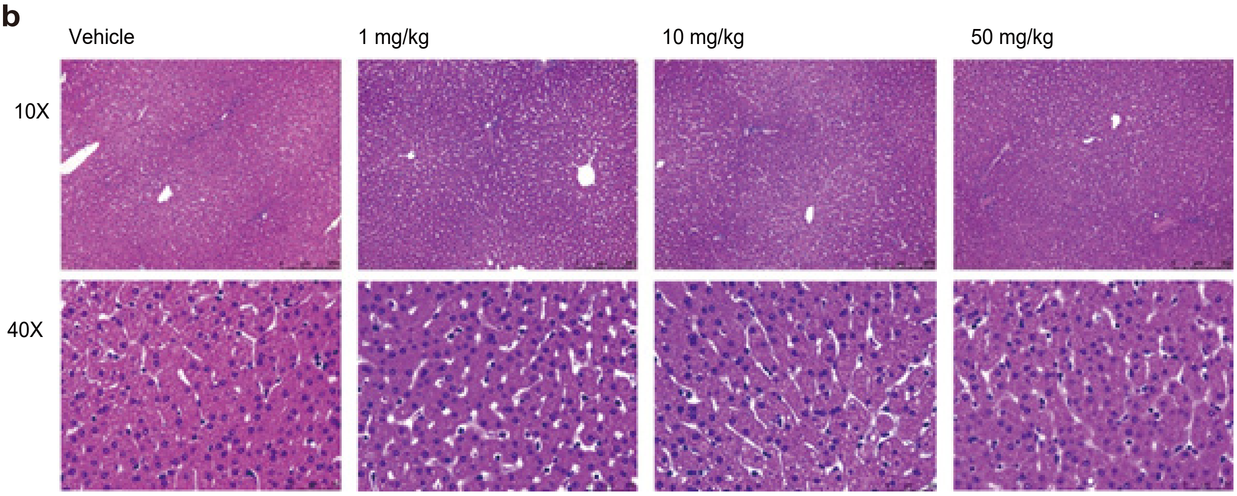

Supplementary Fig. 6

a Blood concentration (left) and calculated PK parameters (right) following a single intravenous infusion of 10 mg/kg IBI319 into non-tumour-bearing C57BL/6-hPD-1/hCD137 mice. n = 3 or 9 (the last collection point) per group, mean and SD. **b** Representative H&E staining of liver tissue from cynomolgus monkeys in the repeated-dose toxicity study. n = 10 animals per group from one experiment were analyzed and the entire slide section of each animal was scanned with microscope. The image of each group shows a representative area of the entire scan**.** No inflammation was found after five doses of IBI319 at different concentrations, indicating its safety in the liver. The magnification is indicated at the left.

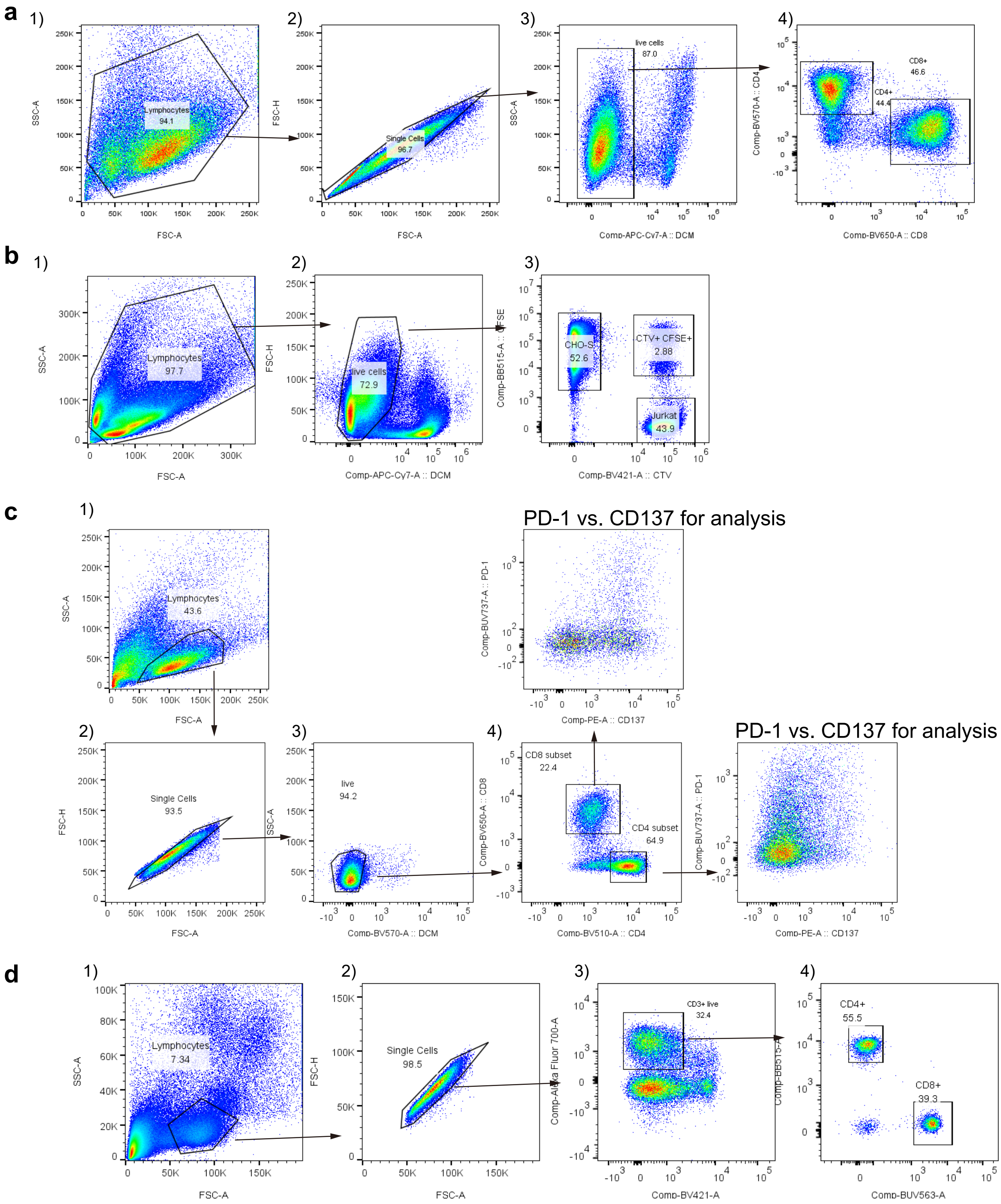
a
1)
Lymphocytes
94.1
SSC-A
FSC-A
2)
Single Cells
96.7
FSC-H
FSC-A
3)
live cells
87.0
SSC-A
Comp-APC-Cy7-A :: DCM
4)
CD8+
46.6
CD4+
44.4
Comp-BV570-A :: CD4
Comp-BV650-A :: CD8
b
1)
Lymphocytes
97.7
SSC-A
FSC-A
2)
live cells
72.9
FSC-H
Comp-APC-Cy7-A :: DCM
3)
CHO-S
52.6
CTV+ CFSE+
2.88
Jurkat
43.9
Comp-BB515-A :: CFSE
Comp-BV421-A :: CTV
c
1)
Lymphocytes
43.6
SSC-A
FSC-A
PD-1 vs. CD137 for analysis
Comp-BUV737-A :: PD-1
Comp-PE-A :: CD137
2)
Single Cells
93.5
FSC-H
FSC-A
3)
live
94.2
SSC-A
Comp-BV570-A :: DCM
4)
CD8 subset
22.4
CD4 subset
64.9
Comp-BV650-A :: CD8
Comp-BV510-A :: CD4
PD-1 vs. CD137 for analysis
Comp-BUV737-A :: PD-1
Comp-PE-A :: CD137
d
1)
Lymphocytes
7.34
SSC-A
FSC-A
2)
Single Cells
98.5
FSC-H
FSC-A
3)
CD3+ live
32.4
Comp-Alexa Fluor 700-A
Comp-BV421-A
4)
CD4+
55.5
CD8+
39.3
Comp-BB515-A
Comp-BUV563-A

Supplementary Fig. 7 Gating strategies for flow cytometry analysis

a Gating strategy for T cell binding assays (Fig. 1e): 1) SSC vs. FSC gating to exclude debris; 2) FSC-H vs. FSC-A gating to exclude doublets; 3) dead cell marker (DCM) vs. SSC-A gating to exclude dead cells; CD4 (BV570) vs. CD8 (BV650) gating to get CD4+ CD8- and CD4-CD8+ cells as the analyzed populations. **b** Gating strategy for cell line binding assays (Fig. 1f, Supplementary Fig. 1e): 1) SSC vs. FSC gating to exclude debris; 2) FSC-H vs. FSC-A gating to exclude doublets; 3) dead cell marker (DCM) vs. FSC-H gating to exclude dead cells; 4) CTV vs. CFSE gating to get CTV+ CFSE+ cells as the analyzed population. **c** Gating strategy for analyzing the expression of PD-1 and CD137 on CHO, Jurkat or PBMC – derived T cell (Supplementary Fig. 2a, c, d): 1) SSC vs. FSC gating to exclude debris; 2) FSC-H vs. FSC-A gating to exclude doublets; 3) dead cell marker (DCM) vs. SSC-A gating to get the live cells as the analyzed population for Jurkat or CHO cells. 4) CD4 (BV510) vs. CD8 (BV650) gating to get CD4+ CD8- and CD4-CD8+ cells as the analyzed populations for PBMC – derived T cell. **d** Gating strategy for analyzing the expression of PD-1 and CD137 on mouse T cells (Supplementary Fig. 3f, g). 1) SSC vs. FSC gating to exclude debris; 2) FSC-H vs. FSC-A gating to exclude doublets; 3) dead cell marker (DCM) vs. CD3 (AF700) gating to get alive CD3 T cells. 4) CD4 (BB515) vs. CD8 (BUV563) gating to get CD4+ CD8- and CD4-CD8+ cells as the analyzed populations.

Dose	Sample ID	Day1	Day 8	Day 15	Day 29	Day 36
0.1 mg/kg	1F001	/	/	1:640	1:10240	1:40960
	1F002	/	/	1:640	1:2560	1:10240
	1F003	/	/	1:10240	1:5120	1:10240
	1M001	/	/	1:640	1:2560	1:10240
	1M002	/	1:40	1:10240	1:20480	1:40960
	1M003	/	/	1:2560	1:320	1:640
	ADA positive rate	0/6	1/6	6/6	6/6	6/6
1 mg/kg	2F001	/	/	1:2560	1:5120	1:10240
	2F002	/	/	1:2560	1:20480	1:40960
	2F003	/	/	1:640	1:2560	1:5120
	2M001	/	/	1:2560	1:1280	1:1280
	2M002	/	/	1:5120	1:5120	1:5120
	2M003	/	1:40	1:10240	1:20480	1:40960
	ADA positive rate	0/6	1/6	6/6	6/6	6/6
10 mg/kg	3F001	/	/	1:2560	1:81920	1:163840
	3F002	/	/	/	1:20480	1:40960
	3F003	/	/	1:320	1:40960	1:40960
	3M001	/	/	1:2560	1:2560	1:40960
	3M002	/	/	1:2560	1:20480	1:40960
	3M003	/	/	1:2560	1:10240	1:40960
	ADA positive rate	0/6	0/6	5/6	6/6	6/6

Supplementary Table 1

The ADA titres of individual cynomolgus monkeys (n = 3/sex/group) in the one-dose PK study. The appearance of ADAs started approximately two weeks after dosing.

Recognized antigen	Fluorophore	Clone name	Catalogue number	Provider	Dilution
**Antibodies for FACS**					
human CD4	BV570	RPA-T4	300534	BD	1:400
human CD4	BV510	UKT4	317444	Biolegend	1:400
human CD8	BV650	RPA-T8	563821	BD	1:400
human CD137	PE	4B4-1	555956	BD	1:50
human PD-1	BUV737	EH12.1	612791	BD	1:200
human PD-1	PE	4B4-1	555956	BD	1:200
human IgG Fc	PE	M1310G05	409304	Biolegend	1:200
mouse CD45	PE-Cy7	30-F11	410311	Biolegend	1:200
mouse CD4	AF488	RM4-5	100529	Biolegend	1:200
mouse CD3	AF700	17A2	100216	Biolegend	1:200
mouse CD8a	BUV563	53-6.7	748535	BD	1:200
mouse NK1.1	APC-Cy7	PK136	47-5941-82	Invitrogen	1:100
mouse CD11b	BUV395	M1/70	563553	BD	1:400
**Antibodies for IHC**					
mouse CD45	-	D3F8Q	70257	Cell Signaling Technology	1:600
mouse F4/80	-	D2S9R	70076	Cell Signaling Technology	1:5000

Supplementary Table 2

Antibody list for flow cytometry and IHC.
