# Supplementary material for: Cancer immune therapy with PD-1-dependent CD137 co-stimulation provides localized tumour killing without systemic toxicity
Source: Nat Commun. 2021 Nov 4;12:6360. doi: 10.1038/s41467-021-26645-6 (PMC8569200; doi:10.1038/s41467-021-26645-6)
Supplement: Supplementary file 3 — Reporting Summary [file 41467_2021_26645_MOESM3_ESM.pdf]

nature portfolio

Corresponding author(s): Wei Xu, Xuan Wang

Last updated by author(s): Sep 13, 2021

Reporting Summary

Nature Portfolio wishes to improve the reproducibility of the work that we publish. This form provides structure for consistency and transparency in reporting. For further information on Nature Portfolio policies, see our Editorial Policies and the Editorial Policy Checklist.

Statistics

For all statistical analyses, confirm that the following items are present in the figure legend, table legend, main text, or Methods section.

n/a	Confirmed	
☐	☒	The exact sample size (*n*) for each experimental group/condition, given as a discrete number and unit of measurement
☐	☒	A statement on whether measurements were taken from distinct samples or whether the same sample was measured repeatedly
☐	☒	The statistical test(s) used AND whether they are one- or two-sided<br>*Only common tests should be described solely by name; describe more complex techniques in the Methods section.*
☒	☐	A description of all covariates tested
☒	☐	A description of any assumptions or corrections, such as tests of normality and adjustment for multiple comparisons
☐	☒	A full description of the statistical parameters including central tendency (e.g. means) or other basic estimates (e.g. regression coefficient) AND variation (e.g. standard deviation) or associated estimates of uncertainty (e.g. confidence intervals)
☐	☒	For null hypothesis testing, the test statistic (e.g. *F*, *t*, *r*) with confidence intervals, effect sizes, degrees of freedom and *P* value noted<br>*Give P values as exact values whenever suitable.*
☒	☐	For Bayesian analysis, information on the choice of priors and Markov chain Monte Carlo settings
☒	☐	For hierarchical and complex designs, identification of the appropriate level for tests and full reporting of outcomes
☒	☐	Estimates of effect sizes (e.g. Cohen's *d*, Pearson's *r*), indicating how they were calculated

Our web collection on statistics for biologists contains articles on many of the points above.

Software and code

Policy information about availability of computer code

Data collection	Flow cytometry and QIFITKIT quantification: FACSDiva software v9.1 (BD); ELISA: SoftMax Pro on SpectraMax i3 (Molecular Devices); RNA sequencing: bcl2fastq v2.20 (Illunima); Luciferase reporter system: SoftMax Pro on SpectraMax i3 (Molecular Devices); SPR: Biacore T200 v3.1 (GE Healthcare); BLI: Fortebio data Acquisition v7.0 (Sartorius); IHC image scan: Aperio Versa v1.0.4.125 (Leica).
Data analysis	Plots and statistics: Graphpadprism v8 (GraphPad Software); Flow cytometry data analysis and display: Flowjo v10.1 (BD); IHC image quatification: HALO v3.3 (indica labs); SPR: Biacore T200 v3.1 (GE Healthcare); BLI: Fortebio data Anaylsis v11.0 (Sartorius); Single cell sequencing data: R language v4.1 (R Core Team). CellRanger (10X Genomics, v2.1.1) analysis pipeline was used for sample demultiplexing, barcode processing, and gene counting based on a mouse genome reference sequence (GRCm38). Seurat (v3.1.2) (https://satijalab.org/seurat/) was used for quality control and downstream analysis. No custom code was generated in this study.

For manuscripts utilizing custom algorithms or software that are central to the research but not yet described in published literature, software must be made available to editors and reviewers. We strongly encourage code deposition in a community repository (e.g. GitHub). See the Nature Portfolio guidelines for submitting code & software for further information.

Data

Policy information about availability of data

All manuscripts must include a data availability statement. This statement should provide the following information, where applicable:

- Accession codes, unique identifiers, or web links for publicly available datasets
- A description of any restrictions on data availability
- For clinical datasets or third party data, please ensure that the statement adheres to our policy

The source data of plots in figures are provided in Source Data file. The single cell RNA sequencing data generated in this study have been deposited in figshare.com

under the hyperlink of https://figshare.com/s/af514391833ca9622e27. All other data generated during the current study are available from the corresponding authors upon reasonable requests.

Field-specific reporting

Please select the one below that is the best fit for your research. If you are not sure, read the appropriate sections before making your selection.

☒ Life sciences ☐ Behavioural & social sciences ☐ Ecological, evolutionary & environmental sciences

For a reference copy of the document with all sections, see nature.com/documents/nr-reporting-summary-flat.pdf

Life sciences study design

All studies must disclose on these points even when the disclosure is negative.

Sample size	No statistical methods were used to predetermine sample size. Sample sizes were determined based on prior experience with similar experiments, or based on pilot experiments. For experiments involving in vitro T cell activation , n=3 healthy donors was chosen as the minimal number. For in vivo efficacy experiments, n=5 mice per group was determined as the minimal sample size. For IHC staining, FACS analysis and ALT/AST measurements of ex vivo samples, n=3 mice per group was chosen as the minimal sample size. n=5/sex for GLP toxicity study, n=3/sex for pharmacokinetic study.
Data exclusions	Tumour size data were excluded from two animals as the mice died before the experiment end point, due to experiment operation.
Replication	All experiments except the in vivo studies were repeated at least twice. All replication were successful. For in vivo studies, the efficacies of studied antibodies and antibody combinations were reproduced in two mouse strains (BALB/c and C57BL/6) baring different tumor models (MC38 and CT26), respectively. At least 5 mice per group was used ensure reproducibility of a same treatment. For IHC staining, FACS analysis and ALT/AST measurements of ex vivo samples, at least 3 mice per group were measured/imaged to ensure the reproducibility of the same treatment within one experiment.
Randomization	Animals were assigned randomly to experimental and control groups. Random animals of each group were chosen for IHC staining, FACS analysis and ALT/AST measurements. For experiments other than animal studies, samples were allocated into experimental and control groups randomly.
Blinding	The investigators were not blinded during data collection and outcome assessment, as no subjective assessments were included. Quantifications were performed using formulas or software processes applied equally to all conditions.

Reporting for specific materials, systems and methods

We require information from authors about some types of materials, experimental systems and methods used in many studies. Here, indicate whether each material, system or method listed is relevant to your study. If you are not sure if a list item applies to your research, read the appropriate section before selecting a response.

Materials & experimental systems

n/a	Involved in the study	
☐	☒	Antibodies
☐	☒	Eukaryotic cell lines
☒	☐	Palaeontology and archaeology
☐	☒	Animals and other organisms
☒	☐	Human research participants
☒	☐	Clinical data
☒	☐	Dual use research of concern

Methods

n/a	Involved in the study	
☒	☐	ChIP-seq
☐	☒	Flow cytometry
☒	☐	MRI-based neuroimaging

Antibodies

Antibodies used	The antibodies used for flow cytometry are listed according to the marker recognized (fluorophore clone name, provider, CatLog number, dilution): hCD4 (BV570, RPA-T4, BD, 300534, 1:400), hCD4 (BV510, UKT4, Biolegend, 317444, 1:400), hCD8 (BV650, RPA-T8, BD, 563821, 1:400), hCD137 (PE, 4B4-1, BD, 555956, 1:50), hPD-1 (BUV737, EH12.1, BD, 612791, 1:200), mCD45 (PE-Cy7, 30-F11, BioLegend, 410311, 1:200), mCD4 (AF488, RM4-5, BioLegend, 100529, 1:200), mCD3 (AF700, 17A2, BioLegend, 100216, 1:200), mCD8a (BUV563, 53-6.7, BD, 748535, 1:200), mNK1.1 (APC-Cy7, PK136, Invitrogen, 47-5941-82, 1:100), mCD11b (BUV395, M1/70, BD, 563553, 1:400), hIgG Fc (PE, M1310G05 BioLegend 409304, 1:200), hPD-1 (PE, 4B4-1, BD, 555956, 1:200) (for QIFIKIT analysis). The antibodies used for IHC are listed according to the marker recognized (clone name, provider, CatLog number, dilution): mCD45 (D3F8Q, Cell Signaling Technology, 70257, 1:600), mF4/80 (D2S9R, Cell Signaling Technology, 70076, 1:5000).
Validation	All antibodies in this study were commercially purchased and have been validated by the vendors for species and application. Validation data are available from the respective vendor's respective websites.

Eukaryotic cell lines

Policy information about cell lines

Cell line source(s)	Jurkat-CD137-NFκB-Luc (human, J2332, Promega), Jurkat-PD-1-NFAT luciferase (human, J1252, Promega), CHOK1-PD-L1 (hamster, J1252, Promega), CHO-S (hamster, A1155701, ThermoFisher Scientific), Jurkat (human, ATCC, TIB-152), MC38 (mouse, OBio), CT26 (mouse, CRL-2638, ATCC); Expi293F(human, A14527, ThermoFisher Scientific).
Authentication	None of the cell lines have been authenticated in this study.
Mycoplasma contamination	All cell lines in this study were tested and negative for mycoplasma contamination.
Commonly misidentified lines (See ICLAC register)	No commonly misidentified cell lines were used in this study.

Animals and other organisms

Policy information about studies involving animals; ARRIVE guidelines recommended for reporting animal research

Laboratory animals	1- Mus musculus, BALB/c, 6-8 weeks old, female (BALB/cJGpt-Pdcd1em1Cin(hPDCD1) Cd274tm1Cin(hCD274)Tnfrsf9em1Cin(hTNFRSF9)/Gpt, T007056)<br>2- Mus musculus, C57BL/6, 5-6 weeks old, female (C57BL/6-Pdcd1tm1(PDCD1)Tnfrsf9tm1(TNFRSF9)/Bcgen, 120516)<br>3- Cynomolgus monkeys, 3-5 years old, male and female.<br><br>Mice were housed in the animal centre of Innovent Biologics with specific pathogen-free(SPF) housing condition (light/dark cycle: 12h/12h; temperature: 22-23°C; humidity: 55%).
Wild animals	No wild animals were used in this study.
Field-collected samples	No field collected samples were used in this study.
Ethics oversight	All mouse-related experiments were approved by the Animal Use and Care Committee of Innovent Biologics. All cynomolgus monkey-related experiments were conducted at WestChina-Frontier PharmaTech Co. , Ltd. (WCFP) in accordance with standard operating procedure and were complied with relevant ethical regulations. The experiments were approved by the Animal Care and Use Committee of WCFP. The 4-week repeated-dose toxicology study was performed in compliance with the principles of national medical products administration (NMPA), food and drug administration (FDA) and organization for economic cooperation and development (OECD) good laboratory practice (GLP).

Note that full information on the approval of the study protocol must also be provided in the manuscript.

Flow Cytometry

Plots

Confirm that:

☒ The axis labels state the marker and fluorochrome used (e.g. CD4-FITC).

☒ The axis scales are clearly visible. Include numbers along axes only for bottom left plot of group (a 'group' is an analysis of identical markers).

☒ All plots are contour plots with outliers or pseudocolor plots.

☒ A numerical value for number of cells or percentage (with statistics) is provided.

Methodology

Sample preparation	For FACS staining, cells were transferred to a 96-well V-bottom plate (2×105 cells/well). Each well was then re-suspended with 50 µL of FACS buffer (PBS with 2% FBS and 2 mM EDTA) containing primary antibodies or CFSE/CTV dye and LIVE/DEAD fixable dead cell staining reagent, and the plate was incubated at 4°C for 20 min. I If necessary, a secondary antibody in FACS buffer was added for 20 min at 4°C. Cells were fixed in 2% Formaldehyde before analysis.
Instrument	FACSymphony A3 (BD Biosciences)
Software	Data were collected using DIVA. Data were analyzed in FlowJo.
Cell population abundance	Cell population abundance is not relevant as no cell sorting was done for this study.
Gating strategy	Gating strategies for flow cytometry analysis:<br>a Gating strategy for T cell binding assays (Fig. 1e): 1) SSC vs. FSC gating to exclude debris; 2) FSC-H vs. FSC-A gating to exclude doublets; 3) dead cell marker (DCM) vs. SSC-A gating to exclude dead cells; CD4 (BV570) vs. CD8 (BV650) gating to get CD4+ CD8- and CD4-CD8+ cells as the analyzed populations.<br><br>b Gating strategy for cell line binding assays (Fig. 1f, Supplementary Fig. 1e): 1) SSC vs. FSC gating to exclude debris; 2) FSC-H

vs. FSC-A gating to exclude doublets; 3) dead cell marker (DCM) vs. FSC-H gating to exclude dead cells; 4) CTV vs. CFSE gating to get CTV+ CFSE+ cells as the analyzed population.

c Gating strategy for analyzing the expression of PD-1 and CD137 on CHO, Jurkat or PBMC – derived T cell (Supplementary Fig. 2a, c, d): 1) SSC vs. FSC gating to exclude debris; 2) FSC-H vs. FSC-A gating to exclude doublets; 3) dead cell marker (DCM) vs. SSC-A gating to get the live cells as the analyzed population for Jurkat or CHO cells. 4) CD4 (BV510) vs. CD8 (BV650) gating to get CD4+ CD8- and CD4-CD8+ cells as the analyzed populations for PBMC – derived T cell.

d Gating strategy for analyzing the expression of PD-1 and CD137 on mouse T cells (Supplementary Fig. 3f, g). 1) SSC vs. FSC gating to exclude debris; 2) FSC-H vs. FSC-A gating to exclude doublets; 3) dead cell marker (DCM) vs. CD3 (AF700) gating to get alive CD3 T cells. 4) CD4 (BB515) vs. CD8 (BUV563) gating to get CD4+ CD8- and CD4-CD8+ cells as the analyzed opulations.

☒ Tick this box to confirm that a figure exemplifying the gating strategy is provided in the Supplementary Information.
